# FEASIBILITY AND ACCEPTABILITY OF A TELEHEALTH-BASED GROUP INTERVENTION FOR VETERANS WITH MCI

**DOI:** 10.1093/geroni/igae098.3978

**Published:** 2024-12-31

**Authors:** Katherine Luci, Patricia Pilkinton, Deanna Dragan, M Lindsey Jacobs, Hannah Apostolou, Allyshia Hinton, Jamie Tedder, Teresa Granger

**Affiliations:** Salem VA Healthcare System, Salem, Virginia, United States; Tuscaloosa VA Medical Center, Tuscaloosa, Alabama, United States; Salem VAMC, Salem, Virginia, United States; The University of Alabama, Tuscaloosa, Alabama, United States; University of Alabama, Northport, Alabama, United States; Tuscaloosa Research Education and Advancement Corporation, Tuscaloosa, Alabama, United States; James H. Quillen VA Medical Center, Johnson City, Tennessee, United States; Tuscaloosa VA Medical Center, Tuscaloosa, Alabama, United States

## Abstract

MInds Navigating the Diagnosis of Mild Cognitive Impairment (MiND-MCI) is a nine-session telehealth intervention developed for older Veterans experiencing stress related to MCI. MIND-MCI targets memory and thinking abilities, stress/coping, and lifestyle risk factors through peer support, education, and skills training. This DoD-funded study aims to evaluate the feasibility, acceptability, and preliminary effectiveness of MIND-MCI to improve QoL among Veterans with MCI. At baseline, participants (n = 16) in this pilot study were ages 61-79 years old and scored in the mild cognitive impairment range (M = 22.8) on the Montreal Cognitive Assessment. Retention rates (88%) and attendance (M = 7/9 sessions) of study completers suggest feasibility and acceptability of the intervention. Eleven participants demonstrated high engagement (attended ≥ 75% of sessions). Participants reported a high level of acceptability (120/147 total score possible) on the Telehealth Usability Questionnaire, and a majority of participants indicated that they agree or completely agree with the acceptability of the intervention on two additional measures (Acceptability of Intervention Measure and the Intervention Appropriateness Measure). Preliminary feedback was obtained from study interventionists to identify barriers and facilitators for delivering this treatment over telehealth. Themes included: range of severity of MCI across participants, session interruptions and delays related to troubleshooting technological issues, strategies used to support older adults’ use of a telehealth platform, and the importance of peer support. Preliminary results support strong engagement with and acceptability of telehealth modalities in this older adult population, a population often presumed to lack telehealth capability and proficiency.

